# Endothelial Glycocalyx: The Missing Link Between Angiogenic Imbalance in Preeclampsia and Systemic Inflammation in HELLP Syndrome

**DOI:** 10.1002/cph4.70032

**Published:** 2025-08-01

**Authors:** Anthony Atallah, Marie‐Nathalie Sarda, Catherine McCarey, Jérôme Massardier, Cyril Huissoud

**Affiliations:** ^1^ Department of Maternal‐Fetal Medicine Hospices Civils de Lyon, Hôpital Femme Mère Enfant, Groupement Hospitalier Est Bron France; ^2^ Claude Bernard University Lyon 1 Lyon France; ^3^ CarMeN Lab—IRIS Team INSERM U1060, INRA, Université Claude Bernard Lyon‐1, INSA‐Lyon, Univ‐Lyon Bron France; ^4^ Department of Immunology Hospices Civils de Lyon, Centre Hospitalier Lyon Sud Pierre‐Benite France; ^5^ Geneva University Hospital Geneva Switzerland

**Keywords:** endothelium, gestational hypertensive disorder, glycocalyx, HELLP syndrome, pathophysiology, preeclampsia, pregnancy

## Abstract

The pathophysiology of preeclampsia and HELLP syndrome relies on systemic vascular endothelial dysfunction, resulting from angiogenic imbalance due to abnormal uteroplacental vascular remodeling and placental ischemia/reperfusion. Recent studies demonstrated that HELLP syndrome falls within the spectrum of secondary microangiopathy due to abnormal complement activation. However, to date, the link between angiogenic imbalance, endothelial dysfunction, and complement activation remains unclear. Building upon current understanding of complement regulation, this paper proposes a novel pathophysiological approach, suggesting a new understanding of HELLP syndrome and preeclampsia, including the undebatable role of sFlt‐1/PlGF and the knowledge of maternal systemic endothelial and renal diseases. We hypothesize that endothelial glycocalyx may be the missing link between angiogenic factors, inflammatory regulation, and endothelial maternal lesions. Targeting the glycocalyx‐endothelium axis may enable novel therapeutic strategies that delay delivery and reduce maternal‐neonatal morbidity in preeclampsia and HELLP syndrome.

## Graphical abstract

Endothelial glycocalyx may be the missing link between angiogenic factors, inflammatory regulation, and endothelial maternal dysfunction in early‐onset preeclampsia and HELLP syndrome.

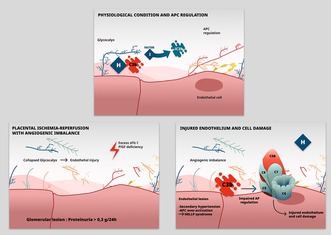

## 1 Introduction

Controversy surrounds the question of whether HELLP is a separate disorder or a severe form of preeclampsia (Berhan 2016). HELLP syndrome (hemolysis, elevated liver enzymes, and low platelets) is a severe diagnosis that may be associated with preeclampsia. Its pathogenesis remains unclear, especially when it occurs before the onset of preeclampsia or without gestational hypertensive disorder (Berhan 2016). Up to 20% of HELLP patients do not demonstrate hypertension or proteinuria (Berhan 2016). The prevalence of maternal and fetal morbi‐mortality in the setting of HELLP syndrome is increased (Fitzpatrick et al. 2014). Untreated, disseminated intravascular coagulopathy is responsible for most maternal complications, followed by hepatic rupture and bleeding (Audibert et al. 1996). Preeclampsia can be expectantly managed until 34 weeks, but HELLP syndrome needs to be resolved by urgent delivery, either after antenatal corticosteroids completion or upon diagnosis (Report of the American College of Obstetricians and Gynecologists' Task Force on Hypertension in Pregnancy 2013). Since delivery is the treatment of choice, HELLP syndrome and severe preeclampsia are the leading causes of induced prematurity worldwide, especially when delivery occurs before 34 weeks (Fanaroff et al. 2007).

To date, the pathophysiologies of preeclampsia and HELLP syndrome remain unclear (Tsatsaris et al. 2008). From general statements about the complement and its regulation, we highlight in this paper a new pathophysiological approach, suggesting a new understanding of HELLP syndrome and preeclampsia, including the undebatable evidence of angiogenic imbalance and the knowledge of maternal systemic endothelial and renal diseases.

## 2 The Complement: Between Innate and Adaptive Immune System

**FIGURE 1:**
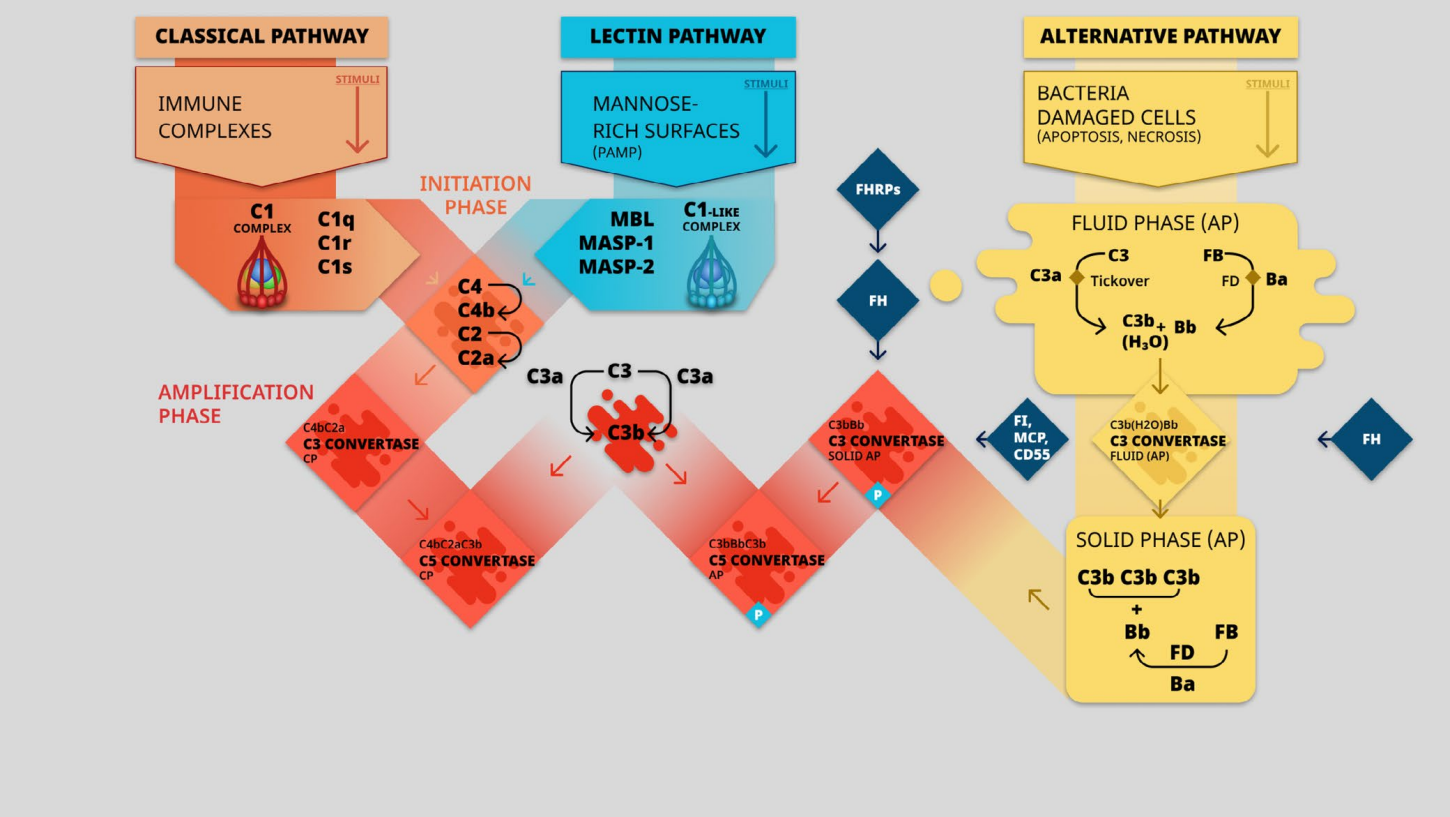
Complement activation pathways. AP, alternative pathway; CP, classical pathway; FB, complement factor B; FD, complement factor D; FH, complement factor H; FHRP, factor‐H related protein; FI, complement factor I; MASP‐1, MBL‐associated serine proteases 1; MASP‐2, MBL‐associated serine proteases 2; MBL, mannose‐binding lectin; MCP, membrane co‐factor protein; P, properdin; PAMP, pathogen associated molecular patterns.

The complement is a vital component of the human immune system, playing a critical role in host defense against pathogens, surveillance, and tissue homeostasis (Girardi et al. 2020). Complement comprises over 50 soluble and membrane‐bound proteins that facilitate the removal of micro‐organisms, damaged cells, and immune complexes. Activation of complement can be triggered through multiple pathways: the classical pathway, the lectin pathway, and the alternative pathway (Vaught et al. 2022) (Figure 1).

– The classical pathway is usually triggered by the binding of immunoglobulin G and M antibodies to antigens on pathogens or foreign cells. This binding initiates a cascade of events, leading to the activation of C1, a complex composed of C1q, C1r, and C1s. Subsequently, activated C1 cleaves complement component C4 and C2, forming the classical C3 convertase (C4bC2a). This convertase cleaves C3 into C3a and C3b, initiating downstream complement effector functions (Vaught et al. 2022).
– The lectin pathway is activated by the recognition of pathogen‐associated molecular patterns (PAMP) on the surface of microorganisms. Pattern recognition molecules (PRM) such as mannose‐binding lectin (MBL), ficolins, and collectins bind to PAMP, triggering the association with MBL‐associated serine proteases (MASP). This association leads to MASP activation and subsequent cleavage of complement components C4 and C2, generating the lectin C3 convertase (C4b2a), which initiates downstream complement activation (Vaught et al. 2022).
– The alternative pathway of complement (APC) is the innate arm of complement activation and functions as a continuous surveillance system throughout physiological conditions. It accounts for > 75% of complement activation products (Harboe and Mollnes 2008). The homeostasis of the APC is dependent upon the balance between APC amplification and APC degradation. After a spontaneous hydrolysis of C3 (C3 tick‐over) resulting in the formation of C3b, C3b is allowed to bind to nearby surfaces (cells, pathogens…) through its ThioEster Domain (TED) (Perkins et al. 2014). In APC amplification setup, Factor B (CFB) binds to C3b and is cleaved by factor D to generate APC C3 convertase (C3bBb). The APC C3 convertase cleaves C3 to generate additional C3b. This phenomenon is called the APC amplification loop (Harboe and Mollnes 2008). APC C3 convertase is assembled and stabilized thanks to Properdin, the only positive regulator of the complement system (Michels et al. 2019).

**FIGURE 2:**
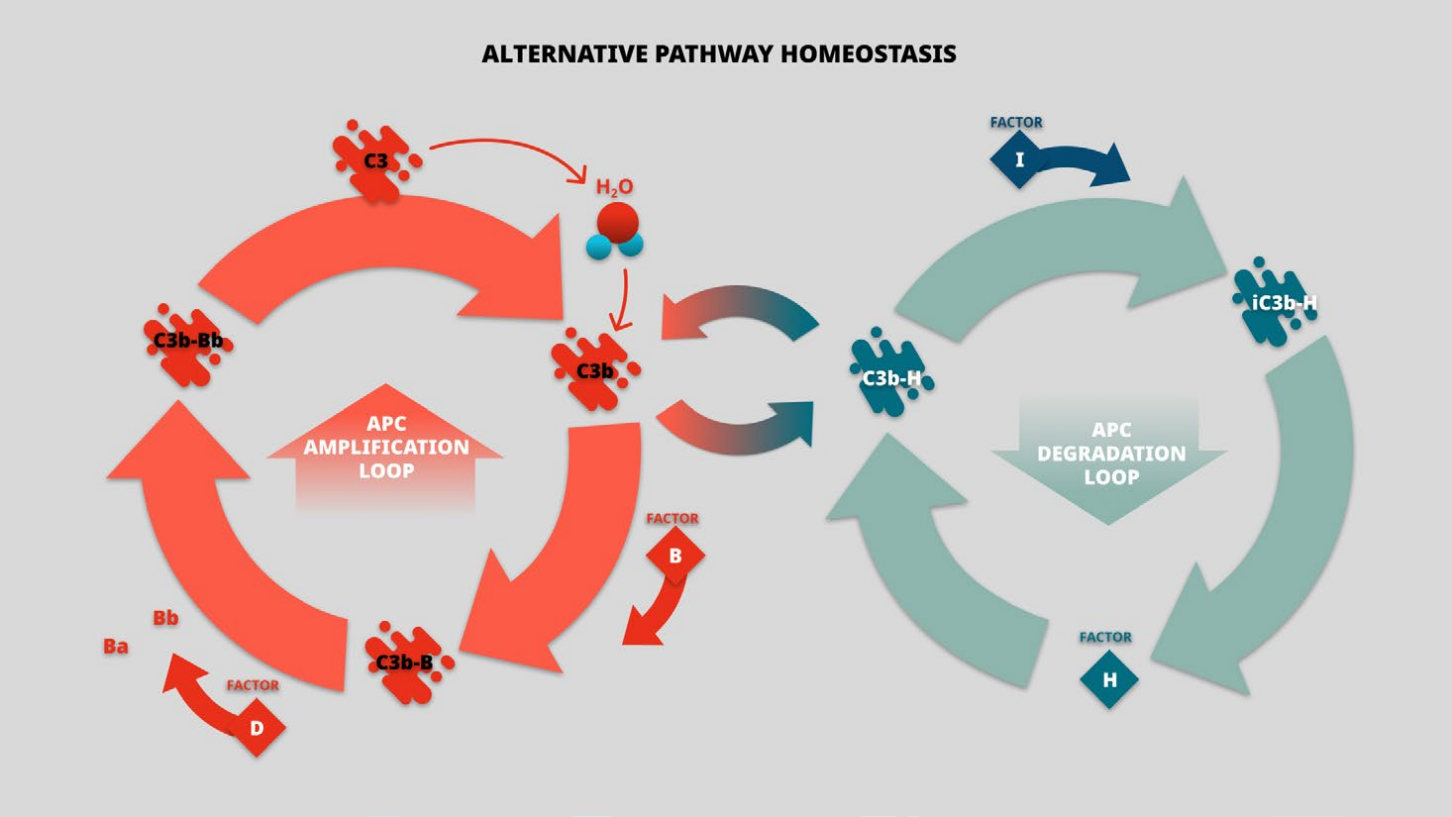
Alternative pathway homeostasis. APC, alternative pathway of complement.

In APC degradation setup, Factor H (CFH) and its co‐factor, Factor I, inhibit C3 amplification and compete with CFB for binding C3b (Vaught et al. 2022) (Figure 2).

**FIGURE 3:**
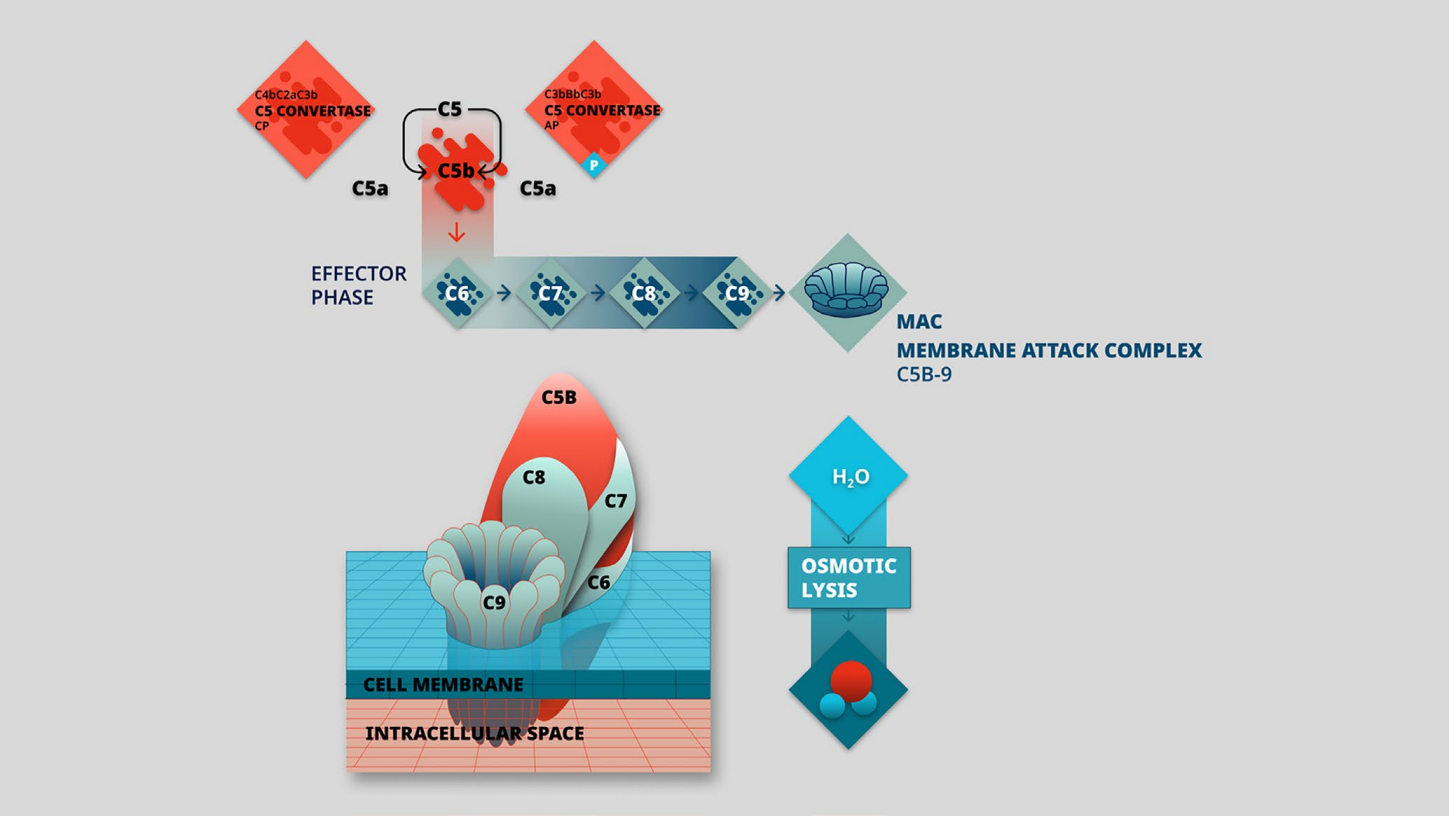
Effector phase of complement pathways. AP, alternative pathway; CP, classical pathway; MAC, membrane attack complex; P, properdin.

These three acknowledged pathways converge to form stable C3 convertase that cleaves C3 into C3a and C3b. The latter triggers the formation of C5 convertases, leading to C5 cleavage into C5a and C5b. C5a exerts pro‐inflammatory properties and C5b oligomerizes with C6, C7, C8, and several C9 proteins to form a cytolytic effector called the membrane attack complex (MAC; C5b‐9) (Zununi Vahed et al. 2021). MAC forms pores in the membrane of pathogens or targeted cells, leading to direct membrane toxicity and osmotic cytolysis (Figure 3).

## 3 Complement Regulation

Complement is regulated by multiple proteins, whose balance is required to avoid over‐activation or immune impairment. Failure to regulate complement can unmask an underlying, to date silent, condition, such as atypical hemolytic uremic syndrome (Saad et al. 2016; Fang et al. 2008). In the meantime, complement upregulation during pregnancy can trigger chronic autoimmune disease such as rheumatoid arthritis, lupus, and anti‐phospholipid syndrome (Chighizola et al. 2020; Kim et al. 2018).

APC regulators can either be soluble serum regulators or membrane regulators.

Complement Factor H (CFH) is the principal negative regulator of the alternative pathway (Vaught et al. 2022). CFH binds to healthy host cell surfaces via bivalent and co‐operative binding mechanisms to cell glycocalyx (e.g., heparan sulfate (HS)) (Perkins et al. 2014). When these binding sites are related to the surface of damaged host cells or pathogens, the C3b‐CFH interaction is compromised, leading to APC amplification. Otherwise, after detecting healthy glycocalyx in host cells, CFH binds C3b and exerts inhibitory effects on the C3 amplification loop by dislocating C3 convertase and preventing C3b and CFB interaction (Vaught et al. 2022). In a physiological state, C3 and CFH are abundant complement proteins in plasma. In the case of acquired or innate CFH deficiency, APC over‐activation results in serious host vulnerability to complement‐mediated attacks (Liszewski et al. 1996).

Complement Factor I (CFI) is another negative regulator of the APC. Its function requires a prior activation of its enzymatic state, after encountering cofactors such as CFH. Once activated, CFI cleaves and deactivates C3b into iC3b, making the liaison to CFB impossible and preventing the APC amplification from occurring (Fremeaux‐Bacchi et al. 2004).

CD46, also called the membrane co‐factor protein MCP, is a transmembrane cell surface protein that serves as a cofactor for the CFI‐mediated cleavage of C3b. Membrane‐bound complement inhibitor CD46 thus inhibits the activity of C3 convertase (Vaught et al. 2022).

CD55 and CD59 are also widely expressed glycophosphatidylinositol anchor membrane proteins that protect cells from complement activation by blocking the cleavage of C3 and C5 and blocking C9 enrollment, respectively.

## 4 The Complement System in Normal Pregnancy

In recent years, numerous studies have shed light on the implications of complement in placental development. The maternal‐fetal interface is rich in complement inhibitors, preventing adverse pregnancy outcomes by protecting the semi‐allogenic graft, promoting immune tolerance, and favoring fetal growth (Pierik et al. 2020).

The complement is involved in multiple processes, ranging from the preimplantation phase to parturition (Girardi et al. 2020).

Complement activation products, such as C3a and C5a, also called anaphylatoxins, have been shown to play a significant role in promoting trophoblast invasion (Regal et al. 2015). Trophoblast cells express complement receptors, such as complement receptor 1 (CR1) and complement receptor 3 (CR3). The binding of complement component to these receptors on trophoblast cells influences various cellular functions, including migration, invasion, and immune modulation (Girardi et al. 2006). Furthermore, complement membrane‐bound regulators have been involved in the regulation of spiral artery remodeling, a critical step in establishing adequate blood flow and shear stress in the placenta (Renaud et al. 2011). Indeed, studies have demonstrated the expression of CD55 and MCP on trophoblast cells and endothelial cells of spiral arteries (Renaud et al. 2011). It must also be noted that anaphylatoxins play a fundamental role during placental development, as they are known to increase vascular permeability, promote angiogenesis, and stimulate smooth muscle contractions (Schumacher et al. 1991). Normal pregnancy is associated with activation of the plasma complement system as determined by increased maternal plasma C3a, C4a, and C5a concentrations. These concentrations are stable throughout the pregnancy and do not change with advancing gestational age, between 20 weeks and term (Richani et al. 2005).

In addition to complement involvement in placental development, complement activation plays a crucial role in maternal endothelial physiology. Complement establishes a reciprocal interaction with the endothelium that also contributes to the secretion of complement components (Fischetti and Tedesco 2006).

Physiologically, it is now clear that the endothelium is not solely a physical barrier between the intravascular and the extravascular sections. Its function includes preventing coagulation by providing an antithrombotic surface. This surface is maintained by HS and glycocalyx proteoglycans present in the matrix around the cells (Bernfield et al. 1999), by the endothelial expression of thrombomodulin and tissue factor inhibitor (Broze 1995). In a homeostatic state, the endothelium secretes complement negative regulators such as C1‐inhibitor, CFH, CFI, CD55, MCP, and CD59 (Fischetti and Tedesco 2006).

## 5 Implication of the Complement in HELLP Syndrome

Recent studies have hypothesized HELLP syndrome may fall within the spectrum of APC diseases and thus, thrombotic microangiopathies (Fakhouri et al. 2020). These studies explored gene sequencing and functional complement assays in HELLP syndrome (Vaught et al. 2016, 2018), highlighting a 50% prevalence of germline variants in genes regulating the APC or positive functional complement assays in patients with HELLP syndrome (Vaught et al. 2016).

Genetic explorations in patients with HELLP syndrome, with or without preeclampsia, and without clinical evidence for atypical Hemolytic Uremic Syndrome (aHUS), revealed mutations in APC regulator such as CFI, MCP, and numerous CFH receptor mutations (CFHR1, CFHR3, CFHR5) (Vaught et al. 2018, 2022; Huerta et al. 2018). Fang et al. (2008) reported a mutation in MCP (CD46) featuring an alanine‐to‐valine substitution at position 304, in the transmembrane region of the protein. Vaught et al. (2016) demonstrated a trend toward significantly increased complement‐mediated cell killing in individuals with classic HELLP syndrome compared to those with severe preeclampsia, using the modified Ham test, proving the complement signature in HELLP syndrome, regardless of the severity of preeclampsia.

There is a significant relationship between C3a concentrations in early pregnancy and the development of HELLP and hypertensive disease later in pregnancy (Lynch et al. 2011). Furthermore, many studies underlined a relationship between complement activation fragment Bb and preeclampsia (Lynch et al. 2008). Activated fragment Bb is increased in the maternal and umbilical venous blood in cases of HELLP and severe preeclampsia when compared with normotensive controls (Hoffman et al. 2014).

Finally, although C5b‐9 has been found to be increased in diseases with excess complement activation such as aHUS, recent studies found no significant difference in serum C5b‐9 levels among participants with severe preeclampsia with or without HELLP and normal controls (Vaught et al. 2016). It must be noted, however, that C5b‐9 detection is not specific to APC. HELLP syndrome presented a higher correlation with tissular C5b‐9 deposition than plasma quantification of C5b‐9 in patient sera (Blakey et al. 2023; Noris et al. 2014). Nevertheless, multiple studies suggest the use of eculizumab, a targeted inhibitor of complement protein C5, in HELLP syndrome and report short‐term clinical and biological improvement (Saad et al. 2016; Vaught et al. 2016; Burwick and Feinberg 2013).

In view of these elements and the level of evidence provided, there is no doubt that complement is involved in the pathophysiology of HELLP syndrome. However, as these proteins of the alternative complement pathway are also involved in aHUS, there seems to be a biological specificity, beyond the mandatory gravid context, in HELLP and preeclampsia.

Hence, we hypothesize that the activation or dysregulation of APC during HELLP syndrome is triggered and/or maintained by the angiogenic imbalance that is pathognomonic of HELLP syndrome and preeclampsia. Thus, we underline the relevance of exploring the link between angiogenic factors and complement dysregulation to understand the biology of HELLP syndrome and hope to reach new therapeutic approaches in the future.

## 6 Pathophysiological Signature for Differential Diagnosis

The description of circulating angiogenic factors as a biological signature in preeclampsia has led to major progress for both diagnosis and prognosis (Verlohren et al. 2022).

Anti‐angiogenic factor soluble fms‐like tyrosine kinase 1 (sFlt‐1) and pro‐angiogenic factor placental growth factor (PlGF) are routinely measured in plasma and serum of individuals for prediction or primary prevention of preeclampsia worldwide (Levine et al. 2004). Dosages are reported as a ratio, which is correlated to the onset of preeclampsia (Levine et al. 2004). In symptomatic pregnant individuals, biological exploration showed an early decrease of PlGF serum levels and a significant rise in sFlt‐1 levels up to 5 weeks prior to preeclampsia onset (Nikuei et al. 2020). In a population of pregnant individuals after 20 weeks of gestation, the sFlt‐1/PlGF ratio demonstrated a high negative predictive value in ruling out the onset of preeclampsia within 7 days among individuals with suspected preeclampsia (Verlohren et al. 2022). Indeed, with a negative predictive value of 99.3% (95% confidence interval, 97.9 to 99.9), sFlt‐1/PlGF ≤ 38 rules out the diagnosis of gestational hypertensive disorder or HELLP syndrome. Thus, in the setting of thrombocytopenia, renal failure, nephropathy, or elevated liver enzymes, or when faced with the differential diagnosis between atypical HELLP syndrome, aHUS, thrombotic thrombocytopenic purpura, nocturnal paroxysmal hemoglobinuria, or any suspicion of systemic microangiopathy, the sFlt‐1/PlGF ratio reduces diagnostic imprecisions and helps to identify the right therapeutic approach (Verlohren et al. 2022; Zeisler et al. 2016; Young et al. 2010; Béranger et al. 2023).

The mechanism of preeclampsia and HELLP syndrome relies on systemic vascular endothelial dysfunction, resulting from abnormal uteroplacental vascular remodeling and placental ischemia/reperfusion (Atallah et al. 2017). Placental ischemia leads to excessive release of sFlt‐1, triggering an angiogenic imbalance due to its binding to the receptor‐binding domains of PlGF (Venkatesha et al. 2006). Some studies hypothesized that in HELLP syndrome, angiogenic imbalance results in endothelial dysfunction with secondary complement activation. However, to date, the link between angiogenic imbalance and APC activation remains unclear (Matsuyama et al. 2021).

## 7 Correlation Between Angiogenic Imbalance and Complement Activation

The association between PlGF and the complement system was evaluated in human umbilical vein endothelial cells (HUVEC). When HUVECs are treated with PlGF, CFH mRNA expression, CFH secretion, and cell viability are higher than in untreated HUVECs, providing the first evidence for an association between angiogenic factors and the inhibitory regulation of the complement system. When HUVECs are treated with both sFlt‐1 and PlGF, CFH mRNA expression, CFH secretion, and cell viability are similar to the ones measured in untreated controls (Matsuyama et al. 2021). Plus, transfection of HUVECs with CFH‐siRNA seems to alter cell viability in comparison to control cells (Matsuyama et al. 2021).

PlGF inhibits C5b‐9 formation on HUVECs since C5b‐9 staining for the measurement of complement activation was lower on HUVECs treated with PlGF in comparison to untreated HUVECs or HUVECs treated with both angiogenic factors (PlGF and sFlt‐1) (Matsuyama et al. 2021).

Therefore, PlGF conferred resistance to complement activation and increased cell viability, indicating a significant protective role against endothelial dysfunction, lesions, or death.

Matsuyama et al. promoted in their work the theory that an angiogenic imbalance in the setting of a preeclampsia/HELLP syndrome, involving increased sFlt‐1 and decreased PlGF, may lower CFH levels, resulting in complement dysregulation, an increase in endothelial C5b‐9 deposition and generating persistent endothelial dysfunction (Matsuyama et al. 2021).

## 8 Discussion: The Endothelial Glycocalyx, a Hypothetical Link Between Angiogenic Imbalance, Endothelial Dysfunction, and Complement Activation

**FIGURE 4:**
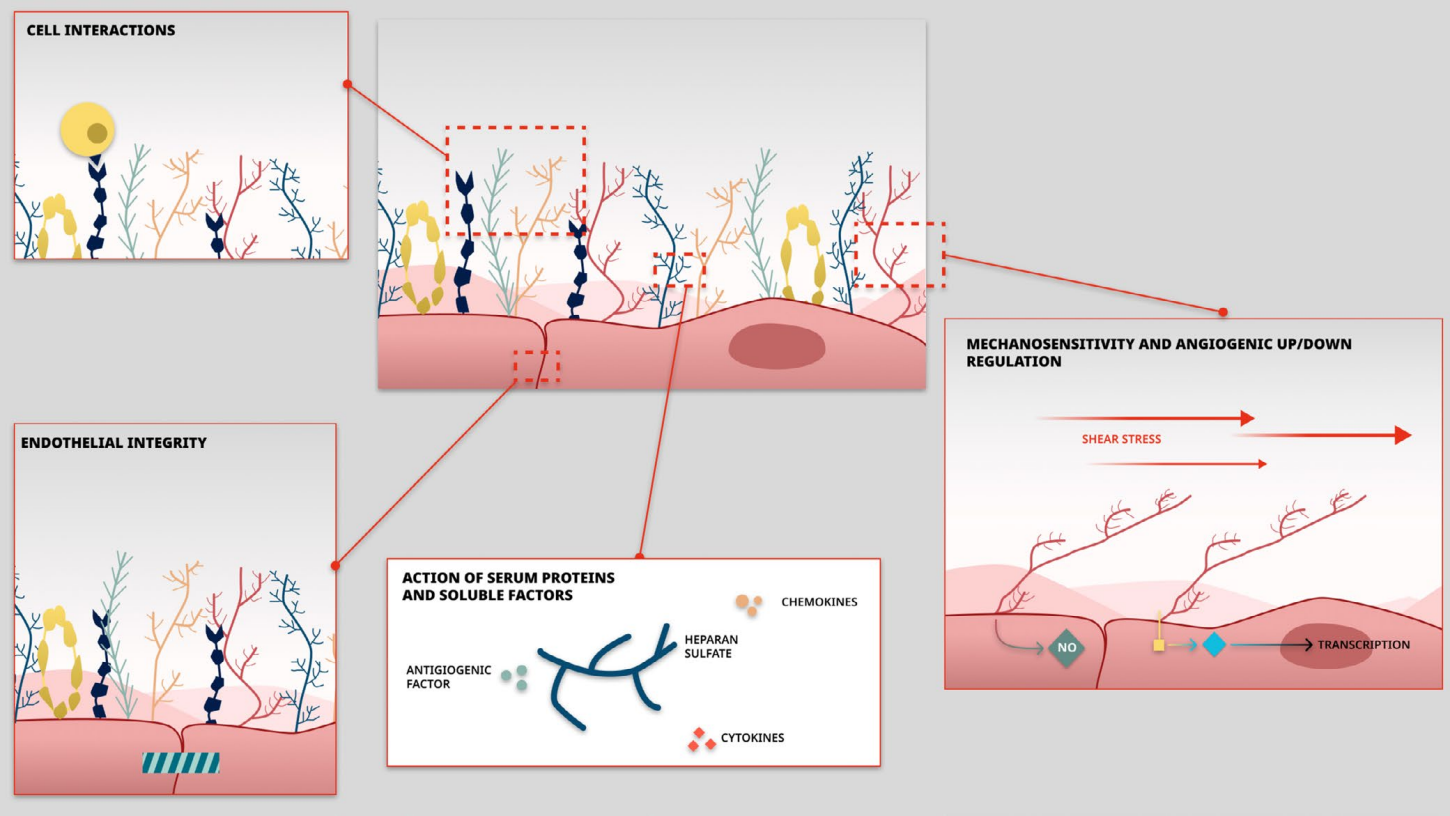
Role of glycocalyx in endothelium physiology. NO, nitric oxide pathway.

The glycocalyx is a non‐uniform, complex structure composed of glycosaminoglycans (such as heparan sulfate), proteoglycans (such as syndecans and glypicans) and glycoproteins (Hu et al. 2021). The glycocalyx is a physiological structure that stands at the interface between the endothelium and the circulating blood, and in the setting of pregnancy, sits between syncytiotrophoblasts micro‐villi brush borders and maternal blood (Fabre‐Gray et al. 2018). It is involved in the pathophysiology of multiple diseases, as a regulator of vascular tone and mecano‐transduction (shear stress) (Atallah et al. 2017), vascular integrity, nitric oxide production (eNOS), angiogenesis, cell interactions, and inflammation (Hu et al. 2021) (Figure 4). It interacts with plasmatic and cellular proteins, chemokines, and growth factors (Milusev et al. 2022). Based on the previously described pathophysiology of preeclampsia (Tsatsaris et al. 2008), endothelial glycocalyx appears to be the common link of the different signaling pathways.

There is clear evidence that CFH is in constant interaction with endothelial heparan sulfate throughout a bivalent and co‐operative binding mechanism (Perkins et al. 2014). This interaction gives CFH its major regulatory function of APC, separating Bb from C3b and serving as cofactor for CFI (Perkins et al. 2014). Indeed, as enunciated previously, CFH is able to discriminate between healthy and damaged host cells through binding sites to glycocalyx (Vaught et al. 2022).

Although sFlt‐1 is a soluble protein, it plays an important role by presenting a binding site for endothelial and placental glycocalyx. It regulates angiogenic cell balance and thus prevents excessive VEGF signaling. In human pregnancy, the placenta is considered to be the main storage site of sFlt‐1 bound to extracellular heparan sulfate (Moore et al. 2020).

Systemic or local inflammatory responses, including the ones triggered by organ ischemia and reperfusion, have been demonstrated to induce rapid loss of glycocalyx and its biological properties (Hu et al. 2021). Previous studies demonstrated excessive glycocalyx degradation among individuals with early‐onset preeclampsia, in the setting of placental ischemia reperfusion, in comparison with non‐preeclamptic pregnant individuals (Weissgerber et al. 2019). Indeed, glycocalyx degradation has been demonstrated by Weissgerber et al. to be associated with endothelial injury in early onset preeclampsia. Women with early onset PE showed a higher glycocalyx degradation, higher plasma concentrations of heparan sulfate proteoglycans and hyaluronic acid, in comparison to normotensive pregnancies (Weissgerber et al. 2019). The authors discuss in their work the assessment of endothelial activation in real time, by using side‐stream dark field imaging of the glycocalyx, providing comprehension into the physiology of early and late‐onset preeclampsia (Weissgerber et al. 2019). Plus, collapsed glomerular glycocalyx (heparan sulfate, syndecans…) seems to be responsible for secondary hypertension (Milusev et al. 2022; Ramnath et al. 2020), significant proteinuria and increase in uremic toxins, that match the symptoms of preeclampsia (Butler et al. 2020; Padberg et al. 2014; Singh et al. 2007; Nieuwdorp et al. 2006). In HELLP syndrome, serum syndecans levels are higher than in gestational age–matched normotensive pregnancies (Hofmann‐Kiefer et al. 2013). Furthermore, excess sFlt‐1 induces a conformational change corresponding to a collapsed glycocalyx with diminished height and increased stiffness (Schulz et al. 2023). A collapsed glycocalyx characterizes a dysfunctional endothelium and favors leukocyte adhesion, monocyte transmigration, decreased cell viability (Schulz et al. 2023), and complement activation (Bongoni et al. 2019; Teoh et al. 2023). Ziganshina et al. (2020) reported in a recent publication that endothelial glycocalyx and its components undergo alteration in the setting of preeclampsia, leading to endothelial activation, stressing the role of endothelial glycocalyx in the pathogenesis of PE.

We hypothesize that preeclampsia and HELLP syndrome are secondary “glycocalyxopathies”, triggered by angiogenic imbalance. In preeclampsia, a collapsed glomerular glycocalyx secondary to excess sFlt‐1 is responsible for proteinuria, which can lead to nephrotic syndrome in severe cases and chronic kidney impairment (Padberg et al. 2014). Systemic damage to the maternal endothelium may cause high blood pressure (Gyarmati et al. 2021). Finally, in certain configurations of the collapsed glycocalyx, activation of the alternative complement pathway through dysregulation of CFH and C3b may explain the development of HELLP syndrome (Teoh et al. 2023).

**FIGURE 5:**
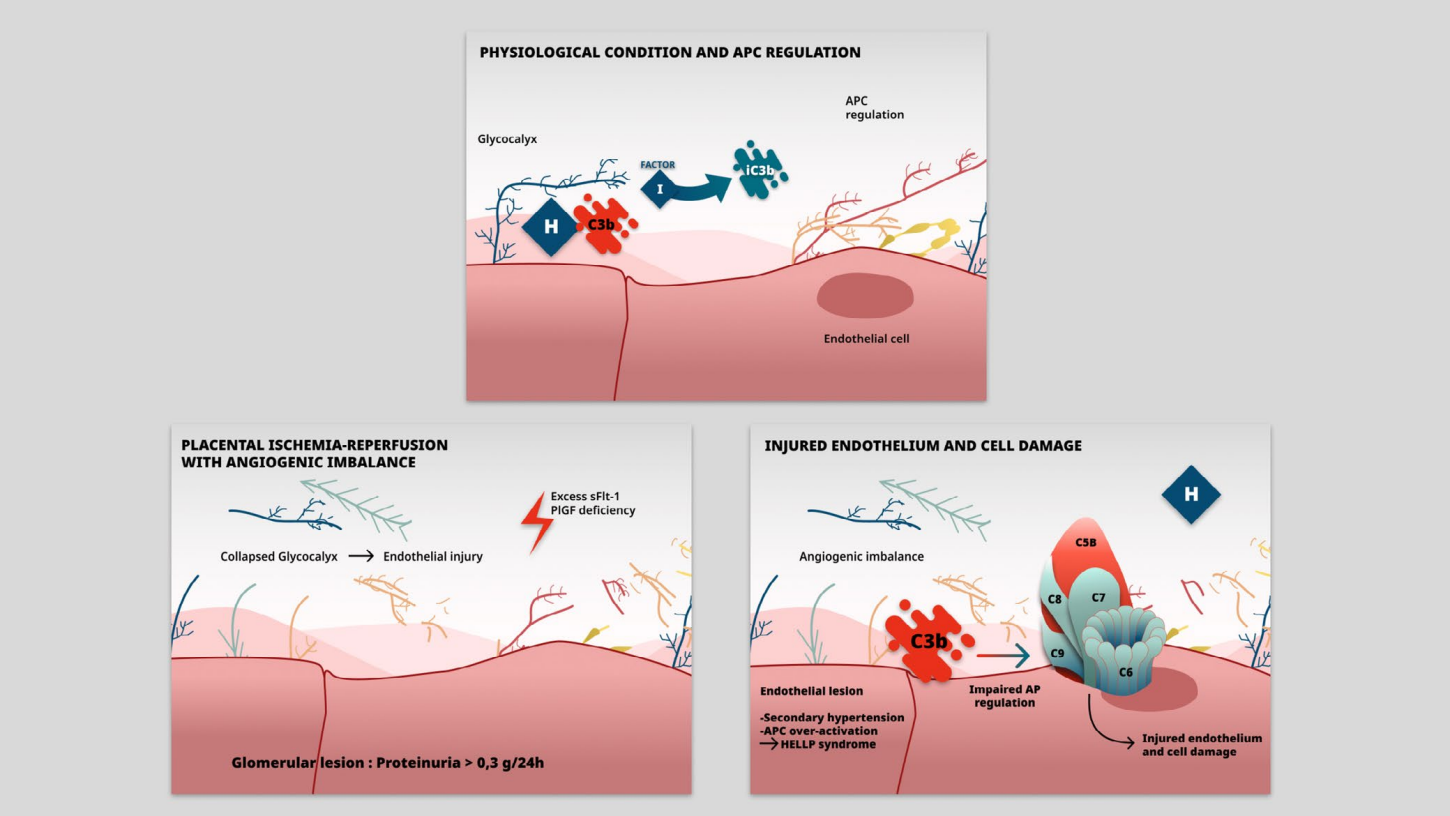
Glycocalyx‐involved pathophysiology of preeclampsia and HELLP syndrome. AP, alternative pathway; APC, alternative pathway of complement; H, complement factor HPlGF, placental growth factor; sFlt‐1, soluble fms‐like tyrosine kinase 1.

The hypothesized pathophysiology of preeclampsia/HELLP syndrome involving angiogenic imbalance and complement activation is summarized in Figure 5.

## 9 Perspective: Immune Implication and New Therapeutic Approaches to Preeclampsia and HELLP Syndrome

The active therapeutic approach in HELLP syndrome is limited to supportive care, expectant management, antihypertensive treatment, and finally delivery (Fakhouri et al. 2020). Since the involvement of complement has been reported in multiple studies, C5 monoclonal antibody eculizumab has been tested to treat HELLP syndrome (Pierik et al. 2020; Burwick and Feinberg 2013; Brocklebank and Kavanagh 2017). Eculizumab is FDA approved for the treatment of paroxysmal nocturnal hemoglobinuria (PNH) and aHUS. In vitro, eculizumab with HELLP serum resulted in increased cell viability (Vaught et al. 2016). In clinical observational studies that reported the use of eculizumab in HELLP syndrome, inhibition of C5 resulted in a reduction of hemolysis, a decrease of liver enzyme levels, a decrease in sFlt‐1, an increase in VEGF, and reduced tissular deposition of C5b‐9 complexes in villous trophoblast cells (Burwick and Feinberg 2013). In the few case reports available in the English literature, delivery was delayed up to 3 weeks after initiation of eculizumab (Burwick and Feinberg 2013). However, despite the unevaluated cost‐effectiveness of eculizumab in HELLP, randomized trials are needed with a validated methodology to provide a therapeutic approach with a solid level of evidence. It must be noted that eculizumab seems to provide a temporary reduction in the inflammatory condition in preeclampsia/HELLP, without any proven effect on angiogenic imbalance, glycocalyx damage, or restoration. To date, no major clinical impacts on endothelial and renal damage, derived from the systemic and placental consequences of angiogenic imbalance, have been studied (Lokki et al. 2020). Secondary hypertension, sodium retention, and nephrotic manifestations are often of severe expression, leading incontestably to delivery (Tsatsaris et al. 2008). Ziganshina et al. (2020) reported that consideration of glycocalyx damage as a key factor of PE might be a relevant approach to prevention, treatment, and rehabilitation of women with early onset preeclampsia. First trimester screening of preeclampsia, sFlt‐1 therapeutic apheresis, and especially glycocalyx understanding should be considered in future research, combining immune modulation, inhibition of glycocalyx damage, and extracellular matrix regeneration to treat preeclampsia and HELLP syndrome (Fischetti and Tedesco 2006). It does however remain to be studied whether glycocalyx‐targeted therapies improve patient health outcomes.

## 10 Conclusion

Preeclampsia is secondary to an angiogenic imbalance triggered by placental ischemia–reperfusion syndrome. In the setting of HELLP syndrome, this imbalance may entail an inflammatory component involving the glycocalyx and the APC. The pathophysiology of HELLP syndrome is that of a pregnancy‐related secondary microangiopathy triggered by sFlt‐1/PlGF imbalance. The dosage of this ratio constitutes a diagnostic signature when differential diagnoses are relevant. Understanding the interplay between glycocalyx, complement, and angiogenic factors is essential to identify potential therapeutic targets to improve the consequences of placental disorders and management of pregnancies with high maternal and neonatal morbidity.

## Author Contributions

Conceptualization: Anthony Atallah. Resources: Anthony Atallah and Jérôme Massardier. Supervision: Jérôme Massardier and Cyril Huissoud. Writing: Anthony Atallah, Marie‐Nathalie Sarda and Catherine McCarey. Review and editing: Anthony Atallah, Marie‐Nathalie Sarda and Catherine McCarey.

## Disclosure

The corresponding author affirms that this manuscript is an honest, accurate, and transparent account of the study being reported; that no important aspects of the study have been omitted; and that any discrepancies from the study as planned have been explained.

## Conflicts of Interest

The authors declare no conflicts of interest.

## Data Availability

The authors have nothing to report.

## References

[cph470032-bib-0001] Atallah, A. , E. Lecarpentier , F. Goffinet , M. Doret‐Dion , P. Gaucherand , and V. Tsatsaris . 2017. “Aspirin for Prevention of Preeclampsia.” Drugs 77, no. 17: 1819–1831. 10.1007/s40265-017-0823-0. 29039130 PMC5681618

[cph470032-bib-0002] Audibert, F. , S. A. Friedman , A. Y. Frangieh , and B. M. Sibai . 1996. “Clinical Utility of Strict Diagnostic Criteria for the HELLP (Hemolysis, Elevated Liver Enzymes, and Low Platelets) Syndrome.” American Journal of Obstetrics and Gynecology 175, no. 2: 460–464. 10.1016/s0002-9378(96)70162-x. 8765269

[cph470032-bib-0003] Béranger, N. , V. Tsatsaris , P. Coppo , A. Veyradier , and B. S. Joly . 2023. “High sFlt‐1 (Soluble Fms‐Like Tyrosine Kinase 1)/PlGF (Placental Growth Factor) Ratio in Pregnancy‐Onset Thrombotic Thrombocytopenic Purpura.” Hypertension 80: e140–e142. 10.1161/HYPERTENSIONAHA.123.20987. 37170814

[cph470032-bib-0004] Berhan, Y. 2016. “Hypertensive Disorder of Pregnancy; No Preeclampsia‐Eclampsia; No Gestational Hypertension; No Hellp Syndrome. Vascular Disorder of Pregnancy Speaks for All.” Ethiopian Journal of Health Sciences 26, no. 2: 177–186. 27222631 10.4314/ejhs.v26i2.12 PMC4864347

[cph470032-bib-0005] Bernfield, M. , M. Götte , P. W. Park , et al. 1999. “Functions of Cell Surface Heparan Sulfate Proteoglycans.” Annual Review of Biochemistry 68: 729–777. 10.1146/annurev.biochem.68.1.729. 10872465

[cph470032-bib-0006] Blakey, H. , R. Sun , L. Xie , et al. 2023. “Pre‐Eclampsia Is Associated With Complement Pathway Activation in the Maternal and Fetal Circulation, and Placental Tissue.” Pregnancy Hypertension 32: 43–49. 10.1016/j.preghy.2023.04.001. 37088032

[cph470032-bib-0007] Bongoni, A. K. , B. Lu , J. L. McRae , et al. 2019. “Complement‐Mediated Damage to the Glycocalyx Plays a Role in Renal Ischemia‐Reperfusion Injury in Mice.” Transplantation Direct 5, no. 4: e341. 10.1097/TXD.0000000000000881. 30993186 PMC6445655

[cph470032-bib-0008] Brocklebank, V. , and D. Kavanagh . 2017. “Complement C5‐Inhibiting Therapy for the Thrombotic Microangiopathies: Accumulating Evidence, but Not a Panacea.” Clinical Kidney Journal 10, no. 5: 600–624. 10.1093/ckj/sfx081. 28980670 PMC5622895

[cph470032-bib-0009] Broze, G. J. 1995. “Tissue Factor Pathway Inhibitor.” Thrombosis and Haemostasis 74, no. 1: 90–93. 8578532

[cph470032-bib-0010] Burwick, R. M. , and B. B. Feinberg . 2013. “Eculizumab for the Treatment of Preeclampsia/HELLP Syndrome.” Placenta 34, no. 2: 201–203. 10.1016/j.placenta.2012.11.014. 23228435

[cph470032-bib-0011] Butler, M. J. , C. J. Down , R. R. Foster , and S. C. Satchell . 2020. “The Pathological Relevance of Increased Endothelial Glycocalyx Permeability.” American Journal of Pathology 190, no. 4: 742–751. 10.1016/j.ajpath.2019.11.015. 32035881 PMC7163249

[cph470032-bib-0012] Chighizola, C. B. , P. A. Lonati , L. Trespidi , P. L. Meroni , and F. Tedesco . 2020. “The Complement System in the Pathophysiology of Pregnancy and in Systemic Autoimmune Rheumatic Diseases During Pregnancy.” Frontiers in Immunology 11: 2084. 10.3389/fimmu.2020.02084. 32973817 PMC7481445

[cph470032-bib-0013] Fabre‐Gray, A. C. M. , C. J. Down , C. R. Neal , R. R. Foster , S. C. Satchell , and V. L. Bills . 2018. “Imaging the Placental Glycocalyx With Transmission Electron Microscopy.” Placenta 74: 59–61. 10.1016/j.placenta.2018.12.004. 30616903

[cph470032-bib-0014] Fakhouri, F. , M. Scully , F. Provôt , et al. 2020. “Management of Thrombotic Microangiopathy in Pregnancy and Postpartum: Report From an International Working Group.” Blood 136, no. 19: 2103–2117. 10.1182/blood.2020005221. 32808006

[cph470032-bib-0015] Fanaroff, A. A. , B. J. Stoll , L. L. Wright , et al. 2007. “Trends in Neonatal Morbidity and Mortality for Very Low Birthweight Infants.” American Journal of Obstetrics and Gynecology 196, no. 2: 147.e1–147.e8. 10.1016/j.ajog.2006.09.014. 17306659

[cph470032-bib-0016] Fang, C. J. , A. Richards , M. K. Liszewski , D. Kavanagh , and J. P. Atkinson . 2008. “Advances in Understanding of Pathogenesis of aHUS and HELLP.” British Journal of Haematology 143, no. 3: 336–348. 10.1111/j.1365-2141.2008.07324.x. 18691170

[cph470032-bib-0017] Fischetti, F. , and F. Tedesco . 2006. “Cross‐Talk Between the Complement System and Endothelial Cells in Physiologic Conditions and in Vascular Diseases.” Autoimmunity 39, no. 5: 417–428. 10.1080/08916930600739712. 16923542

[cph470032-bib-0018] Fitzpatrick, K. E. , K. Hinshaw , J. J. Kurinczuk , and M. Knight . 2014. “Risk Factors, Management, and Outcomes of Hemolysis, Elevated Liver Enzymes, and Low Platelets Syndrome and Elevated Liver Enzymes, Low Platelets Syndrome.” Obstetrics and Gynecology 123, no. 3: 618–627. 10.1097/AOG.0000000000000140. 24499757

[cph470032-bib-0019] Fremeaux‐Bacchi, V. , M. A. Dragon‐Durey , J. Blouin , et al. 2004. “Complement Factor I: A Susceptibility Gene for Atypical Haemolytic Uraemic Syndrome.” Journal of Medical Genetics 41, no. 6: e84. 10.1136/jmg.2004.019083. 15173250 PMC1735822

[cph470032-bib-0020] Girardi, G. , J. J. Lingo , S. D. Fleming , and J. F. Regal . 2020. “Essential Role of Complement in Pregnancy: From Implantation to Parturition and Beyond.” Frontiers in Immunology 11: 1681. 10.3389/fimmu.2020.01681. 32849586 PMC7411130

[cph470032-bib-0021] Girardi, G. , D. Yarilin , J. M. Thurman , V. M. Holers , and J. E. Salmon . 2006. “Complement Activation Induces Dysregulation of Angiogenic Factors and Causes Fetal Rejection and Growth Restriction.” Journal of Experimental Medicine 203, no. 9: 2165–2175. 10.1084/jem.20061022. 16923853 PMC2118387

[cph470032-bib-0022] Gyarmati, G. , C. O. Jacob , and J. Peti‐Peterdi . 2021. “New Endothelial Mechanisms in Glomerular (Patho)biology and Proteinuria Development Captured by Intravital Multiphoton Imaging.” Frontiers in Medicine 8: 765356. 10.3389/fmed.2021.765356. 34722598 PMC8548465

[cph470032-bib-0023] Harboe, M. , and T. E. Mollnes . 2008. “The Alternative Complement Pathway Revisited.” Journal of Cellular and Molecular Medicine 12, no. 4: 1074–1084. 10.1111/j.1582-4934.2008.00350.x. 18419792 PMC3865650

[cph470032-bib-0024] Hoffman, M. C. , K. K. Rumer , A. Kramer , A. M. Lynch , and V. D. Winn . 2014. “Maternal and Fetal Alternative Complement Pathway Activation in Early Severe Preeclampsia.” American Journal of Reproductive Immunology 71, no. 1: 55–60. 10.1111/aji.12162. 24128411 PMC4067768

[cph470032-bib-0025] Hofmann‐Kiefer, K. F. , J. Knabl , N. Martinoff , et al. 2013. “Increased Serum Concentrations of Circulating Glycocalyx Components in HELLP Syndrome Compared to Healthy Pregnancy: An Observational Study.” Reproductive Sciences 20, no. 3: 318–325. 10.1177/1933719112453508. 22872545

[cph470032-bib-0026] Hu, Z. , I. Cano , and P. A. D'Amore . 2021. “Update on the Role of the Endothelial Glycocalyx in Angiogenesis and Vascular Inflammation.” Frontiers in Cell and Developmental Biology 9: 734276. 10.3389/fcell.2021.734276. 34532323 PMC8438194

[cph470032-bib-0027] Huerta, A. , E. Arjona , J. Portoles , et al. 2018. “A Retrospective Study of Pregnancy‐Associated Atypical Hemolytic Uremic Syndrome.” Kidney International 93, no. 2: 450–459. 10.1016/j.kint.2017.06.022. 28911789

[cph470032-bib-0028] Kim, M. Y. , M. M. Guerra , E. Kaplowitz , et al. 2018. “Complement Activation Predicts Adverse Pregnancy Outcome in Patients With Systemic Lupus Erythematosus and/or Antiphospholipid Antibodies.” Annals of the Rheumatic Diseases 77, no. 4: 549–555. 10.1136/annrheumdis-2017-212224. 29371202 PMC6037302

[cph470032-bib-0029] Levine, R. J. , S. E. Maynard , C. Qian , et al. 2004. “Circulating Angiogenic Factors and the Risk of Preeclampsia.” New England Journal of Medicine 350, no. 7: 672–683. 10.1056/NEJMoa031884. 14764923

[cph470032-bib-0030] Liszewski, M. K. , T. C. Farries , D. M. Lublin , I. A. Rooney , and J. P. Atkinson . 1996. “Control of the Complement System.” Advances in Immunology 61: 201–283. 10.1016/s0065-2776(08)60868-8. 8834497

[cph470032-bib-0031] Lokki, A. I. , M. Haapio , and J. Heikkinen‐Eloranta . 2020. “Eculizumab Treatment for Postpartum HELLP Syndrome and aHUS—Case Report.” Frontiers in Immunology 11: 548. 10.3389/fimmu.2020.00548. 32308654 PMC7145984

[cph470032-bib-0032] Lynch, A. M. , R. S. Gibbs , J. R. Murphy , P. C. Giclas , J. E. Salmon , and V. M. Holers . 2011. “Early Elevations of the Complement Activation Fragment C3a and Adverse Pregnancy Outcomes.” Obstetrics and Gynecology 117, no. 1: 75–83. 10.1097/AOG.0b013e3181fc3afa. 21173647 PMC5267353

[cph470032-bib-0033] Lynch, A. M. , J. R. Murphy , T. Byers , et al. 2008. “Alternative Complement Pathway Activation Fragment Bb in Early Pregnancy as a Predictor of Preeclampsia.” American Journal of Obstetrics and Gynecology 198, no. 4: 385–389. 10.1016/j.ajog.2007.10.793. PMC2362503 18221926

[cph470032-bib-0034] Matsuyama, T. , T. Tomimatsu , K. Mimura , et al. 2021. “Complement Activation by an Angiogenic Imbalance Leads to Systemic Vascular Endothelial Dysfunction: A New Proposal for the Pathophysiology of Preeclampsia.” Journal of Reproductive Immunology 145: 103322. 10.1016/j.jri.2021.103322. 33887508

[cph470032-bib-0035] Michels, M. A. H. M. , E. B. Volokhina , N. C. A. J. van de Kar , and L. P. W. J. van den Heuvel . 2019. “The Role of Properdin in Complement‐Mediated Renal Diseases: A New Player in Complement‐Inhibiting Therapy?” Pediatric Nephrology 34, no. 8: 1349–1367. 10.1007/s00467-018-4042-z. 30141176 PMC6579773

[cph470032-bib-0036] Milusev, A. , R. Rieben , and N. Sorvillo . 2022. “The Endothelial Glycocalyx: A Possible Therapeutic Target in Cardiovascular Disorders.” Frontiers in Cardiovascular Medicine 9: 897087. 10.3389/fcvm.2022.897087. 35647072 PMC9136230

[cph470032-bib-0037] Moore, K. H. , H. Chapman , and E. M. George . 2020. “Unfractionated Heparin Displaces sFlt‐1 From the Placental Extracellular Matrix.” Biology of Sex Differences 11, no. 1: 34. 10.1186/s13293-020-00311-w. 32600401 PMC7325113

[cph470032-bib-0038] Nieuwdorp, M. , H. L. Mooij , J. Kroon , et al. 2006. “Endothelial Glycocalyx Damage Coincides With Microalbuminuria in Type 1 Diabetes.” Diabetes 55, no. 4: 1127–1132. 10.2337/diabetes.55.04.06.db05-1619. 16567538

[cph470032-bib-0039] Nikuei, P. , M. Rajaei , N. Roozbeh , et al. 2020. “Diagnostic Accuracy of sFlt1/PlGF Ratio as a Marker for Preeclampsia.” BMC Pregnancy and Childbirth 20, no. 1: 80. 10.1186/s12884-020-2744-2. 32033594 PMC7006116

[cph470032-bib-0040] Noris, M. , M. Galbusera , S. Gastoldi , et al. 2014. “Dynamics of Complement Activation in aHUS and How to Monitor Eculizumab Therapy.” Blood 124, no. 11: 1715–1726. 10.1182/blood-2014-02-558296. 25037630 PMC4162105

[cph470032-bib-0041] Padberg, J. S. , A. Wiesinger , G. S. di Marco , et al. 2014. “Damage of the Endothelial Glycocalyx in Chronic Kidney Disease.” Atherosclerosis 234, no. 2: 335–343. 10.1016/j.atherosclerosis.2014.03.016. 24727235

[cph470032-bib-0042] Perkins, S. , K. W. Fung , and S. Khan . 2014. “Molecular Interactions Between Complement Factor H and Its Heparin and Heparan Sulfate Ligands.” Frontiers in Immunology 5: 126. 10.3389/fimmu.2014.00126. 24744754 PMC3978290

[cph470032-bib-0043] Pierik, E. , J. R. Prins , H. van Goor , et al. 2020. “Dysregulation of Complement Activation and Placental Dysfunction: A Potential Target to Treat Preeclampsia?” Frontiers in Immunology 10: 3098. 10.3389/fimmu.2019.03098. 32010144 PMC6974484

[cph470032-bib-0044] Ramnath, R. D. , M. J. Butler , G. Newman , et al. 2020. “Blocking Matrix Metalloproteinase‐Mediated Syndecan‐4 Shedding Restores the Endothelial Glycocalyx and Glomerular Filtration Barrier Function in Early Diabetic Kidney Disease.” Kidney International 97, no. 5: 951–965. 10.1016/j.kint.2019.09.035. 32037077 PMC7184681

[cph470032-bib-0045] Regal, J. F. , J. S. Gilbert , and R. M. Burwick . 2015. “The Complement System and Adverse Pregnancy Outcomes.” Molecular Immunology 67, no. 1: 56–70. 10.1016/j.molimm.2015.02.030. 25802092 PMC4447554

[cph470032-bib-0046] Renaud, S. J. , T. Cotechini , J. S. Quirt , S. K. Macdonald‐Goodfellow , M. Othman , and C. H. Graham . 2011. “Spontaneous Pregnancy Loss Mediated by Abnormal Maternal Inflammation in Rats Is Linked to Deficient Uteroplacental Perfusion.” Journal of Immunology 186, no. 3: 1799–1808. 10.4049/jimmunol.1002679. 21187445

[cph470032-bib-0047] Report of the American College of Obstetricians and Gynecologists' Task Force on Hypertension in Pregnancy . 2013. “Hypertension in Pregnancy.” Obstetrics and Gynecology 122, no. 5: 1122–1131. 10.1097/01.AOG.0000437382.03963.88. 24150027

[cph470032-bib-0048] Richani, K. , E. Soto , R. Romero , et al. 2005. “Normal Pregnancy Is Characterized by Systemic Activation of the Complement System.” Journal of Maternal‐Fetal & Neonatal Medicine 17, no. 4: 239–245. 10.1080/14767050500072722. 16147832 PMC1421513

[cph470032-bib-0049] Saad, A. F. , J. Roman , A. Wyble , and L. D. Pacheco . 2016. “Pregnancy‐Associated Atypical Hemolytic‐Uremic Syndrome.” AJP Reports 6, no. 1: e125–e128. 10.1055/s-0036-1579539. 26989566 PMC4794438

[cph470032-bib-0050] Schulz, A. , C. C. Drost , B. Hesse , et al. 2023. “The Endothelial Glycocalyx as a Target of Excess Soluble Fms‐Like Tyrosine Kinase‐1.” International Journal of Molecular Sciences 24, no. 6: 5380. 10.3390/ijms24065380. 36982455 PMC10049398

[cph470032-bib-0051] Schumacher, W. A. , J. C. Fantone , S. E. Kunkel , R. C. Webb , and B. R. Lucchesi . 1991. “The Anaphylatoxins C3a and C5a Are Vasodilators in the Canine Coronary Vasculature In Vitro and In Vivo.” Agents and Actions 34, no. 3–4: 345–349. 10.1007/BF01988727. 1810146

[cph470032-bib-0052] Singh, A. , S. C. Satchell , C. R. Neal , E. A. McKenzie , J. E. Tooke , and P. W. Mathieson . 2007. “Glomerular Endothelial Glycocalyx Constitutes a Barrier to Protein Permeability.” Journal of the American Society of Nephrology 18, no. 11: 2885–2893. 10.1681/ASN.2007010119. 17942961

[cph470032-bib-0053] Teoh, C. W. , M. Riedl Khursigara , C. G. Ortiz‐Sandoval , et al. 2023. “The Loss of Glycocalyx Integrity Impairs Complement Factor H Binding and Contributes to Cyclosporine‐Induced Endothelial Cell Injury.” Frontiers in Medicine 10: 891513. 10.3389/fmed.2023.891513. 36860338 PMC9968885

[cph470032-bib-0054] Tsatsaris, V. , T. Fournier , and N. Winer . 2008. “Pathophysiology of Preeclampsia.” Journal de Gynecologie, Obstetrique et Biologie de la Reproduction 37, no. 1: 16–23. 10.1016/j.jgyn.2007.08.003. 18036745

[cph470032-bib-0055] Vaught, A. J. , E. Braunstein , S. Chaturvedi , K. Blakemore , and R. A. Brodsky . 2022. “A Review of the Alternative Pathway of Complement and Its Relation to HELLP Syndrome: Is It Time to Consider HELLP Syndrome a Disease of the Alternative Pathway.” Journal of Maternal‐Fetal & Neonatal Medicine 35, no. 7: 1392–1400. 10.1080/14767058.2020.1755650. 32338085 PMC13317799

[cph470032-bib-0056] Vaught, A. J. , E. M. Braunstein , J. Jasem , et al. 2018. “Germline Mutations in the Alternative Pathway of Complement Predispose to HELLP Syndrome.” JCI Insight 3, no. 6: e99128. 10.1172/jci.insight.99128. 29563339 PMC5926944

[cph470032-bib-0057] Vaught, A. J. , E. Gavriilaki , N. Hueppchen , et al. 2016. “Direct Evidence of Complement Activation in HELLP Syndrome: A Link to Atypical Hemolytic Uremic Syndrome.” Experimental Hematology 44, no. 5: 390–398. 10.1016/j.exphem.2016.01.005. 26921648 PMC4995062

[cph470032-bib-0058] Venkatesha, S. , M. Toporsian , C. Lam , et al. 2006. “Soluble Endoglin Contributes to the Pathogenesis of Preeclampsia.” Nature Medicine 12, no. 6: 642–649. 10.1038/nm1429. 16751767

[cph470032-bib-0059] Verlohren, S. , S. P. Brennecke , A. Galindo , et al. 2022. “Clinical Interpretation and Implementation of the sFlt‐1/PlGF Ratio in the Prediction, Diagnosis and Management of Preeclampsia.” Pregnancy Hypertension 27: 42–50. 10.1016/j.preghy.2021.12.003. 34915395

[cph470032-bib-0060] Weissgerber, T. L. , O. Garcia‐Valencia , N. M. Milic , et al. 2019. “Early Onset Preeclampsia Is Associated With Glycocalyx Degradation and Reduced Microvascular Perfusion.” Journal of the American Heart Association 8, no. 4: e010647. 10.1161/JAHA.118.010647. 30764695 PMC6405679

[cph470032-bib-0061] Young, B. , R. J. Levine , S. Salahuddin , et al. 2010. “The Use of Angiogenic Biomarkers to Differentiate Non‐HELLP Related Thrombocytopenia From HELLP Syndrome.” Journal of Maternal‐Fetal & Neonatal Medicine 23, no. 5: 366–370. 10.1080/14767050903184207. 19701867 PMC3132879

[cph470032-bib-0062] Zeisler, H. , E. Llurba , F. Chantraine , et al. 2016. “Predictive Value of the sFlt‐1:PlGF Ratio in Women With Suspected Preeclampsia.” New England Journal of Medicine 374, no. 1: 13–22. 10.1056/NEJMoa1414838. 26735990

[cph470032-bib-0063] Ziganshina, M. M. , E. L. Yarotskaya , N. V. Bovin , S. V. Pavlovich , and G. T. Sukhikh . 2020. “Can Endothelial Glycocalyx be a Major Morphological Substrate in Pre‐Eclampsia?” International Journal of Molecular Sciences 21, no. 9: 3048. 10.3390/ijms21093048. 32357469 PMC7246531

[cph470032-bib-0064] Zununi Vahed, S. , Y. Rahbar Saadat , and M. Ardalan . 2021. “Thrombotic Microangiopathy During Pregnancy.” Microvascular Research 138: 104226. 10.1016/j.mvr.2021.104226. 34252400

